# Managing Boutonniere and swan-neck deformities

**DOI:** 10.1186/1753-6561-9-S3-A50

**Published:** 2015-05-19

**Authors:** Donald Lalonde

**Affiliations:** 1 Department of Plastic and Reconstructive Surgery, Dalhousie University, Saint John, New Brunswick, Canada, E2K 1J5

## Boutonniere deformities

Surgery is not the answer for boutonniere deformities. I have tried many times without success. I have yet to meet a surgeon who can tell me honestly he has an operation that works well for boutonniere. However, boutonnieres can get good results with splinting, especially with the development of the new relative motion flexion splint by Dr Wyndell Merritt of the USA.

## This is how we successfully treat chronic boutonnieres

1) serial cast them until full or near full extension of the PIP joint is obtained and the DIP joint is able to flex as much as the DIP joint of the same finger on the other hand. The lateral bands are still volar to the axis of the PIP joint if the DIP is still hyperextending.

2) start an 8 week full time (24/7) PIP extension splint. In the splint, have the patient practice active extension of the PIP and active flexion of the DIP

3) After 8 weeks, start them on an 8 week course of:

a) In the day, take them out of full time PIP extension splinting and

b) Put them into a relative motion flexion splint. This splint keeps the affected finger MP joint relatively flexed compared to the other MP joints. It stops the MP joint from hyperextending. MP hyperextension is bad for boutonnieres because it tightens the lateral bands, creating a downward force on them at the PIP level. MP hyperextension also relaxes the lateral slip of the long extensor pull on the lateral bands, which permits a lateral band downward pull at the PIP joint level.

c) Continue nighttime PIP extension splinting.

## Acute boutonnieres

can be put in a relative motion flexion splint (or simulate one with a pencil between the fingers flexing down the boutonniere finger). If the relative flexion of that finger fixes the boutonniere, that is all that is required for 8 weeks. If not, they are treated like chronic boutonnieres.

## Swan neck deformities

Early in the swan neck process, they are flexible. The PIP joint still has full active flexion. We like to treat them with 24/7 swan neck rings as there is no morbidity, they are functional and the rings are made of silver and stylish. Many people use rings instead of having the surgery. The rings are a figure of 8 at the PIP joint that keep the joint in slight flexion, stop full extension, and allow full flexion.

If the patient wants an operation instead, it will take them 2-4 months to recover. I prefer to take a slip of superficialis cut proximally and anchor it into the proximal phalanx to keep the PIP joint in just a little flexion.

Fixed swan neck deformities are best treated in my hands with PIP fusion in a better position of function.

